# Effects of the Attention Training Technique on Auditory Hallucinations in Schizo-Affective Disorder: A Single Case Study

**DOI:** 10.1155/2018/1537237

**Published:** 2018-08-09

**Authors:** Karin E. P. Carter, Adrian Wells

**Affiliations:** ^1^Greater Manchester Mental Health NHS Foundation Trust, UK; ^2^School of Health Sciences, University of Manchester, UK

## Abstract

A 41-year-old female with schizo-affective disorder presenting with an eight-year history of auditory hallucinations participated in a single case treatment study (A-B-A-B-A-C-B) of the effects of the Attention Training Technique (ATT). No antipsychotic medication was prescribed in this case following a serious adverse reaction in the past. The aim of the study was to test the impact of ATT on the frequency and duration of hallucinations using a repeated return to baseline followed by an alternating treatment design. The alternative intervention consisted of autogenic relaxation instructions. The patient monitored the frequency, duration, and her distress over the voices on a daily basis during baseline and intervention phases across a study period of 80 weeks. Visual analysis of the data showed that ATT when introduced at three phases following baselines or control conditions was associated with a reduction in auditory hallucination frequency and duration compared to the other phases. This contrasted with the autogenic relaxation intervention that was associated with an increase in duration and frequency of voices. The perceived benefits of ATT were maintained for varying periods of time.

## 1. Introduction

Auditory hallucinations are a positive symptom for some individuals with a psychotic disorder. Psychological interventions focusing on addressing hallucinations have included behavioural strategies such as distraction, focusing, coping behaviour enhancement, and the combination of cognitive-behavioural approaches focusing on content and meaning of such experiences [1]. The International Consortium on Hallucination Research [2] summarised that combination interventions have shown benefit on positive symptoms, but there remains a paucity of evidence for the effects of individual techniques that directly address auditory hallucinations. The present study aimed to test the effects on auditory hallucinations of the specific technique of attention training.

Deficits of attention and executive functioning have been highlighted as common difficulties in psychotic disorders that may contribute to symptoms [3]. Furthermore, deficits in this domain may disadvantage individuals from benefiting fully from psychosocial interventions. As a consequence remediation interventions such as attention shaping using rewards [4] or drill and practice procedures in cognitive rehabilitation studies [5] have examined the impact on performance and functioning of these patients.

In these and other packages, attentional strategies have been part of multicomponent and multitheoretical approaches, the complex composition of which means it is not possible to identify the active components or separate their individual effects. With this in mind the present study set out to test the possible effects of a stand-alone individual technique that has been found to be effective in treating anxiety and depression. The Attention Training Technique (ATT: [6]) was developed based on the Self-Regulatory Executive Function (S-REF) model of psychological disorder [7]. The basic premise of the S-REF model is that psychological disorders are the result of self-regulatory strategies dominated by perseverative thinking (worry, rumination, and excessive attention to threat, e.g., self-monitoring). The ATT is aimed at enhancing executive control such that these perseverative processes can be moderated effectively by the individual. Consistent with the application of the S-REF model to psychosis and the concomitant application of techniques such as the ATT, the perseverative strategies implicated have been found to be positively associated with psychotic symptoms in a systematic review of 51 studies [8]. These results imply that the ATT which is intended to moderate such processes may be useful in the context of treating psychosis symptoms. In particular, following the ATT individuals may find it easier to disengage from worry, rumination, and threat-focused attention strategies that accompany the experience of hallucinations, and it is these processes that are hypothesised to exacerbate such experiences.

The ATT is a brief intervention requiring participants to listen to an array of sounds and to systematically apply selective attention, attention switching, and divided attention. The rationale for ATT emphasises the development of metacognitive control in which the individual is directed to relate to any spontaneous thoughts or intrusions as they might relate to the sounds in the environment and to control attention as instructed without dealing with or attempting to attenuate such experiences. The technique is explicitly presented not as a distraction from spontaneous thoughts or experiences but as a means of discovering that the control of attention is independent of internal and external events.

In a systematic review of ATT, Knowles, Foden, El-Deredy, and Wells [9] examined its efficacy in clinical and nonclinical samples involving single case studies and randomised trials. They reported large effect sizes (improvement rate difference= 0.74-1.00) for ATT in patients with anxiety disorders (panic disorder, health anxiety, and social anxiety) and depression where single case designs were used, with ATT having a beneficial impact on a variety of outcomes. Knowles et al. [9] also reported medium to large between-group effect sizes favouring ATT over control groups of d=0.52-0.91 for intrusive thoughts and d=0.73 for negative affect. In an independent review of ATT studies, Fergus and Bardeen [10] concluded that ATT could be classified as a “possibly efficacious treatment” based on the available evidence. However, to date only two studies of ATT (both single cases) have been reported with individuals with a diagnosis of schizophrenia. Valmaggia, Boumann, and Schuurman [11] reported that ATT may be helpful with hallucinations. In their case study, a patient with schizophrenia in which hallucinations were the primary source of residual distress received a modified form of ATT over eight sessions. A follow-up at 2 and 6 months reported continued improvement (on the PSYRATS), with the voices reportedly no longer being a source of distress if they occurred. However, this study did not include a no-treatment baseline, in the absence of which the results remain tentative and we cannot infer that they are associated with introducing the ATT.

Levaux, Laroi, Offerlin-Meyer, Danion, and Van der Linden [12] implemented ATT with a patient with paranoid schizophrenia who reported distress with intrusive thoughts (“of a paranoid nature”). An AB (baseline followed by the intervention) design was used. Again a modified form of ATT was administered as the patient was reported to have working memory deficits and the authors wanted to control the pace of learning. The authors reported that ATT was associated with improved positive symptoms as measured by the PANSS and improved scores on a validated measure (Thought Control Ability Questionnaire) of perceived ability to control thoughts, images, and impulses. The gains were maintained at 6 months' follow-up.

So far no published studies have used the original version of the ATT that has been found to be effective in emotional disorders and they have used single case methodologies that had significant design limitations (lack of baseline, no return to baseline, or introduction of alternative treatment) that impact on interpretability.

Given the data suggesting the potential efficacy of stand-alone ATT, but with the limitations of these earlier studies in psychosis we aimed to use more rigorous single case methodology to test the effects associated with the technique. Specifically, we focused on the frequency and duration of auditory hallucinations in a case of schizo-affective disorder. We used a repeated return to baseline design followed by an alternative treatment. Such a “withdrawal” or “reversal” design ([13], p.88) allows for the assessment of effects of the repeated introduction and withdrawal of treatment so that effects can be more reliably interpreted as effects associated with introduction of the intervention itself. We augmented this strategy with introduction of an alternative treatment later in the sequence to allow some additional assessment of the possible contribution of nonspecific treatment factors (e.g., placebo effects).

## 2. Method

### 2.1. Subject

A 41-year-old woman was referred with an eight-year history of “chronic auditory hallucinations.” The diagnosis was recorded as schizo-affective disorder. The patient had previously been diagnosed with paranoid psychosis and a manic-depressive illness had also been queried. The patient had well documented hypomanic phases, she had been prescribed and was taking lithium, and her mood had stabilized. She was also taking thyroxine daily. No antipsychotic medication was prescribed as a consequence of a previous adverse reaction.

## 3. Design

A single case A-B-A-B-A-C-B design was used. Such a repeated return to baseline (i.e., withdrawal) design (A-B-A-B-A) component allows an examination of the effects of repeated introduction and removal of the target intervention. If the treatment (B) has an effect then this should be apparent as an observable change in primary outcomes compared to baseline (A). Demonstration of a reliable effect is determined by replication of the effects over baseline performance over the course of time. Effects are evaluated in terms of characteristics in graphed data such as abrupt changes in level and changes in slope across phases. In the present study we also added a second treatment (autogenic relaxation instructions) to the time series because we wanted to observe possible nonspecific (e.g., placebo) effects that might be associated with the provision of an intervention.

If the treatment ATT had an effect an observable and replicable reduction in frequency and duration of auditory hallucinations seen as a change in level and/or slope would be found. No specific predictions were made as to duration of this effect. With the second intervention the autogenic instructions acted to control for nonspecific factors associated with introducing a credible intervention. This offered a means to assess any impact of nonspecific factors as a reference against which to compare the effects of the ATT.

In summary, two interventions were used in this study. The principal intervention (intervention B) was the Attention Training Technique and the second (intervention C) was a control intervention based on autogenic relaxation instructions.

## 4. Procedure

The therapist met briefly with the patient each week to collect and administer symptom diaries and questionnaires and to practice the planned interventions (B or C). When ATT (time 1) was first introduced the patient was seen 9 times over 3 weeks to practice the technique, 5 times over 2 weeks when ATT was introduced at time 2, and 3 times in one week in order to introduce ATT at time 3. The relaxation intervention was undertaken 3 times before the patient continued with it independently. Each intervention was preceded by a rationale that the patient reported as credible and the interventions were matched for length of session time.

The patient was provided with a written summary of how to undertake ATT, as a recording of the technique was not an option at the time. The means by which the patient learned the technique was by direct practice with the therapist (KC) using attentional focusing on sounds and spatial locations within and outside of her home (e.g., clock, radio white noise, heating system, bird song, and traffic). The patient was required to repeat the technique independently as set homework tasks. Similarly the patient was given a summary of how to undertake the relaxation task and practiced this initially with the therapist (KC) in the home and as follow-up homework.

No cognitive or metacognitive challenging, coping skills training, or other treatment techniques were undertaken. The patient reported a strong conviction that the voices that she heard were external and real individuals; no attempt was made to address these beliefs.

## 5. Measures

The patient monitored the frequency (main outcome) and duration of voices and also rated distress on a daily basis and completed a number of other questionnaires weekly during baseline and intervention phases (the other questionnaires were not intended for graphical analysis and were given less frequently and are not reported here). The patient completed a diary as the primary outcome, recording each episode (frequency) of voices, their duration, and her distress. The distress rating for each episode was made on an 11-point scale where 0 = no distress and 10 = the worst possible distress. These idiosyncratic measures were selected in order to allow for the repeated and efficient collection of specific data of interest.

## 6. Treatment

Attention Training (ATT) was practiced with the patient in each session and lasted approximately 12-15 minutes. A range of sounds were used and there were 6-9 sounds/locations made available or identified at the same time: 3 individual sounds in the room, 3 sounds outside of the room, and 3 sounds in the distance. The therapist when undertaking the training sessions used her voice as one sound and made a tapping noise with a pen on a hard surface. A radio tuned between stations and a kitchen timer was also used to generate sounds. Other natural sounds were available to the patient such as her heating system, the sound of people outside of her rooms, traffic in the near distance, the sound of birds, the wind, and faint traffic sounds in the distance. When the therapist was not present the patient used other sounds in the room. The sounds varied but the patient had a radio and a portable kitchen timer (that ticked) that she could use to generate sounds and opportunistic sounds were present in the environment. In an ATT practice session the therapist gave paced auditory instructions to focus on individual sounds and then practiced switching between them before dividing attention and focusing on all sounds simultaneously.

Relaxation consisted of in session practice of relaxing muscle groups and lasted approximately 12-15 minutes. The patient was asked to relax the muscles in her neck and shoulders and asked to focus on feelings of heaviness and relaxation. She was asked to concentrate on the feelings of heaviness in her limbs and muscle groups which was facilitated by repeating a phrase to herself 5 times “my shoulders are heavy and relaxed, heavy like lead.” This was then repeated with the focus on warmth in that part of the body. This sequence of heaviness and warmth was undertaken with her neck and shoulders, each arm and each leg.

During each treatment phase the patient was instructed to practice ATT or relaxation 2-3 times a day, and an audio recording of the instructions was not provided but written instructions were given. The patient kept a record of homework practice. In general the ATT was practiced between 1 and 3 times a day (more typically 2 times a day) and the relaxation 1-2 times a day.

## 7. Results

**Figure 1:**
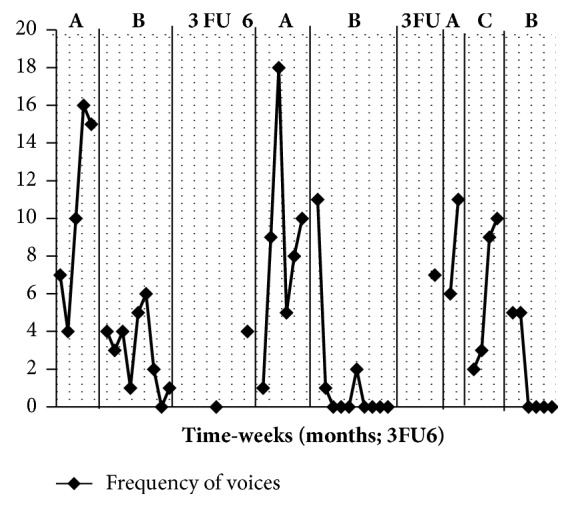
Frequency of voices (y-axis = total number of episodes per week; x-axis = weekly sessions with periods of follow-up (FU) measured at 3 and 6 months after each B phase. Note: A = baseline, B = ATT, and C = autogenics).

**Figure 2:**
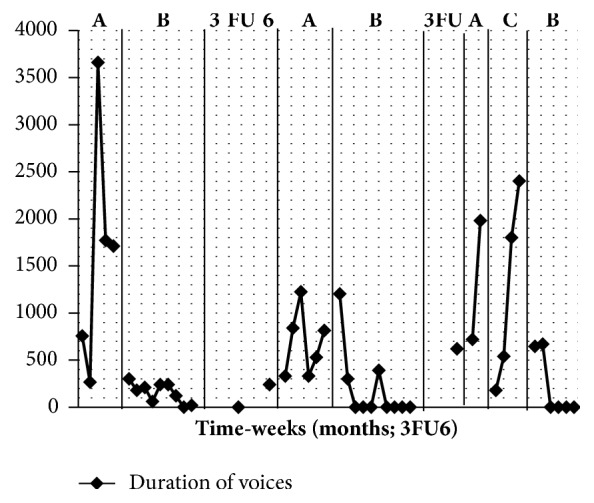
Duration of voices (y-axis = total minutes per week; x-axis = weekly sessions with periods of follow-up (FU) measured at 3 and 6 months after each B phase. Note: A = baseline, B = ATT, and C = autogenics).

The results are presented in Figures 1 and 2. The first baseline phase (A) demonstrates an increase in frequency and duration of voices during the baseline period. Following the introduction of ATT (B) both frequency and duration of symptom measures decreased substantially and the gains appeared to be maintained at 3 and 6 months and during the weekly return to baseline monitoring following this. It is notable that the degree of fluctuation in symptom scores during the return to baseline (A) phase is less pronounced compared with the initial baseline. Reintroduction of the ATT (B) was associated with a further reduction of symptoms which levelled out at zero frequency during this phase. However, the frequency and duration of voices increased again after 3 months and during the weekly return to baseline monitoring that followed. After the introduction of the control treatment (C: autogenic relaxation) there was an immediate reduction in level of symptoms but this was not sustained and the increasing slope shows that they increased again during this phase. This intervention was halted at the patient's request. Attention training was reintroduced at this point. With the reintroduction of the ATT (B) hallucination frequency decreased and became zero which was sustained for the remainder of this phase.

The patient recorded a distress rating for each episode of voices. Distress ratings could be scored from 0 = no distress to 10 = the worst possible distress. Her distress ratings when voices were present ranged from 2 to 10/10. They did not drop below 2 unless there was a total absence of hallucinations at which point the rating dropped to zero. For this individual some level of distress was always present if voices were present.

The patient's self-report of treatment effect was interesting, and she reported not knowing why the voices would stop during treatment. This was not addressed during the study but at the end of the final ATT sequence (i.e., end of the study) an explanation was offered that her experience of voices appeared to be a hallucination linked to her attention rather than to real individuals. This was accepted by her at the time.

## 8. Discussion and Conclusions

The repeated introduction of ATT appeared to be associated with concomitant reductions in hallucination frequency and duration. Changes in level and slope of these variables were evident each time the ATT was introduced compared with baselines and when compared with the control intervention. In contrast the control intervention was associated with an initial reduction followed by an increase in duration and episode frequency. The repeated effects across phases appear to lend support to ATT being the active component in the observed changes. Crucially, these results extend the study of Valmaggia et al. [11] and the effect of ATT as reviewed for emotional disorders [10]. They suggest that the ATT may be associated with reductions in auditory hallucinations and the technique may have applications in the treatment of psychotic symptoms.

Whilst the present study examined the effect of ATT alone, this technique is usually a component of metacognitive therapy (MCT, [14]) and preliminary evidence supports the effects of full MCT in psychosis [15, 16]. Metacognitive therapy differs from CBT approaches such as metacognitive training in that it does not challenge beliefs about voices or modify cognitive distortions in interpretations of events. Metacognitive training can be understood as “an amalgam of cognitive-behavioural therapy (CBT), cognitive remediation (CRT) and psychoeducation” [17]. In contrast, metacognitive therapy [18] and its techniques such as ATT were developed based on a specific information processing model of self-regulation with the specific aim of regulating worry and rumination processes and modifying metacognitive beliefs and executive control.

### 8.1. Limits of the Study

The study was conducted with a single individual and therefore it is unclear whether the results would generalise to others. It would seem worthwhile to explore this with additional participants with auditory hallucinations either as a case series or as a randomised control trial.

The study would also have benefitted from the use of standardised measures so that comparisons can be made between studies in the future. Additional measures could also inform researchers whether there were wider benefits or indirect outcomes of the intervention which were not the direct focus of the work. Further work could also explore how to optimise the duration of the reported benefit suggested in this case study.
